# Distribution of visuo-attentional resources while reading multiple words

**DOI:** 10.1371/journal.pone.0341917

**Published:** 2026-02-02

**Authors:** Valentina Bandiera, Lisa S. Arduino, Roberta Daini, Marialuisa Martelli, Silvia Primativo

**Affiliations:** 1 Human Science Department, LUMSA University, Rome, Italy; 2 Psychology Department, University of Milano-Bicocca, Italy; 3 Don Carlo Gnocchi IRCCS Foundation ONLUS, Milan, Italy; 4 Sapienza University, Rome, Italy; Hong Kong Baptist University, HONG KONG

## Abstract

Recent studies have investigated the role of semantic processing in the parafovea using the Rapid Parallel Visual Presentation (RPVP) paradigm, which involves the simultaneous presentation of two words, one in the fovea (W1) and one in the parafovea (W2). Results have shown that both accuracy and response times are influenced by the semantic relatedness between the words. These findings suggest that semantic information can be extracted from the parafovea and in parallel with the processing of the foveal word. In the present study, we aimed to provide further evidence of parallel semantic processing and to gain deeper insight into the availability and spatial distribution of attentional resources when semantic relatedness is present. Two experiments were conducted. In both, the first part replicated the previous RPVP setup: two words were simultaneously presented and participants were required to read them aloud. Frequency and semantic relatedness between the two words were manipulated. Results replicated previous findings. Crucially, each experiment included a second task: as participants began reading the two words, a probe (an asterisk) could appear to the right of the parafoveal word (Experiment 1) or above the foveal word (Experiment 2). Probe detection times and accuracy were equally facilitated at both positions when the two words was of high-frequency and semantically related. In contrast, when W1 was of low-frequency, neither accuracy nor reaction times in probe detection benefited from parafoveal processing, suggesting that an increased load on the foveal word hinders parafoveal processing. Finally, the same probe detection results were obtained – both in terms of mean reaction times and accuracy – across the two spatial positions, indicating an even distribution of attentional resources across foveal and parafoveal words.

## Introduction

Reading is an everyday activity, yet it remains a highly complex cognitive task that involves multiple components, including linguistic processes (orthography, phonology, lexical and semantic access), visual perception, attention and eye movements. To efficiently extract information, readers move their eyes to bring text into the fovea, the area of the retina with the highest visual acuity. Considerable research has investigated the nature of the information that can be extracted from the parafoveal region [1–3], which in left-to-right reading languages, extends rightwards approximately 15–20 characters beyond the fovea [4]. Despite reduced visual acuity and attentional resources, readers can extract useful information from the parafovea [5,6].

A key debate in the reading literature concerns: i) the serial vs. parallel distribution of attention to multiple words and ii) what types of information (orthographic, phonological, lexical, or semantic) can be extracted from parafoveal vision and how it interacts with foveal processing.

Understanding how attention is allocated and distributed across text during reading is central to both experimental and theoretical research on eye movement control [7]. Cognitive models of reading are often categorized based on how they assume attention is allocated. The Sequential Attention Shift (SAS) models posit that attention shifts serially from one word to the next, tightly linking eye movements to lexical access. Although SAS models allow some parafoveal preview—where readers extract partial information from upcoming words— they assume full lexical processing only occurs once a word is fixated (e.g., E-Z Reader: [8–11]). For example, [12] examined perceptual capacity limits by comparing semantic and color judgments in a dual-task paradigm. Their results seem to indicate that, whereas change detection for low-level non-linguistic features can approach unlimited capacity, semantic judgments exhibit a clear dual-task cost. This pattern could be compatible with serial processing under certain conditions. Similarly, the authors [13] reported the same pattern when using sentences instead of words.

In contrast, parallel models (e.g., SWIFT [14–17]) propose that multiple words can be processed simultaneously. These models suggest that attention is distributed across multiple words in the visual field, with the extent of parallel processing depending on cognitive and linguistic constraints (see for example [15]). Unlike serial models, parallel models assume that multiple words can be processed at the same time, with processing depth decreasing as words move away from fixation [18–20]. This graded allocation of attention explains the parafoveal-on-foveal effect – where parafoveal word properties influence foveal processing (PoF; [21]) often measured through the foveal inspection time [22–24]. Notably, Snell et al. [24], by using a syntactic categorization task within a flanking paradigm, showed that syntactic information can be integrated across foveal and parafoveal words. Similarly, in a semantic categorization task, bilingual participants showed semantic processing of the parafoveal words [25]. Indeed, in both studies, the authors showed that decision response times were influenced by the syntactic and the semantic congruency of flanking words, respectively. Overall, these competing models differ in how much and what kind of information is assumed to be accessible from the parafovea.

Another critical issue concerns the role of foveal load, namely how the cognitive demand associated with processing the foveal word may modulate parafoveal processing. According to the foveal load hypothesis [26], increased processing difficulty in the fovea (e.g., due to low-frequency words) reduces the amount of information that can be extracted from the parafovea. Indeed, Henderson and Ferreira [26] first demonstrated this effect: when the foveal word was more difficult to process (e.g., a low-frequency word), the parafoveal preview benefit (PPE) was reduced compared to an easier-to-process word (e.g., a high-frequency word).

The Foveal Load Effect (FLE) aligns well with serial models, where the processing of the parafoveal word is strongly influenced by the attentional resources required to process the foveal word and, only once this is at least partially processed, attention can move forward to the parafoveal word, through an attentional moving window acting similarly to the spotlight hypothesized by Posner [27]. Conversely, the parallel models posit that the focus of attention can be shifted independently of the eye’s fixation position and allocated to a spatially extended region of the text, to support the processing of multiple words at once. Attention could be narrowed or widened due to a failure or a successful recognition of words [14,15]. Thus, on the basis of serial and parallel models’ assumptions, the foveal load can modulate parafoveal processing in two different ways: affecting the linguistic depth of processing according to serial models [9] and the spatial extent of processing according to the parallel models [28]. However, it should be noted that recent research questioned the robustness of the FLE. For example, although foveal load can influence saccade targeting [29], this does not necessarily reflect reduced parafoveal processing [30]. Replications of the FLE are limited and sometimes contradictory [31], and the effect may vary across writing systems [30,32].

The current study has two aims: i) to replicate prior evidence of fast semantic processing in parafovea with the Rapid Parallel Visual Presentation (RPVP) paradigm [33–35]; and ii) to examine the spatial distribution of attentional resources and any changes due to semantic relatedness by introducing a non-linguistic probe-detection task. In our previous work, we employed the Rapid Parallel Visual Presentation (RPVP) paradigm in Italian, where participants read aloud two simultaneously presented words—one in the fovea and one in the parafovea—that were either semantically related or unrelated. Across different exposure durations, we consistently observed both parafoveal preview benefits (PPE) and PoF effects: participants were more accurate and faster when words were semantically related and when W1 was of high-frequency. These effects occurred even under brief exposure (≈100 ms), suggesting rapid access to semantic information and supporting parallel processing mechanisms [33,34]. Crucially, even under high foveal load (i.e., low-frequency W1), semantic relatedness facilitated foveal reading times, implying that semantic processing of the parafoveal word occurred despite increased cognitive demand.

In the present study, we explored more directly the distribution of visual attention immediately after a two-words reading task (RPVP paradigm). To this end, we adopted a probe detection task, with the probe placed either in the foveal or parafoveal region. To ensure that the stimuli fell on the fovea and parafovea, an eye-tracker was used. If attention is distributed serially, we might observe a good performance for probe detection in all conditions in fovea and an advantage in parafoveal probe detection for semantically related words. On the other hand, if attention is distributed in parallel, we might observe a faster probe detection in case of semantic relatedness between the two words, regardless of whether the probe appears in the foveal or parafoveal location.

## Materials and methods

### Participants

The experimental protocol was approved by the local ethic committee of LUMSA University (CERS) with a protocol number 16/2023. Two different groups of participants from April 1^st^ to December 15^th^ 2024, were recruited for each of the two experiments described below, and all provided written informed consent. Based on a G*power analyses ([36]; power = 0.95, α = 0.05, effect size = 0.6), the required sample size for each experiment was estimated to be N = 32 participants.

All participants self-reported normal or corrected-to-normal vision and no history of dyslexia. In Experiment 1, 32 participants took part (mean age = 22.4 years, range = 20–29 years, SD. = 2.3, 21 females, mean education = 14 years). In Experiment 2, 32 participants were also tested (mean = 21 years, range = 18–25 years, SD. = 2.3, 23 females, mean education = 14 years). All participants were unaware of the purpose of the study.

### Stimuli

Eighty pairs of Italian nouns were selected from the CoLFIS database [37]. All words were nouns 4–5 letters in length (mean = 4.5). Written word frequency (high and low) and semantic relatedness between the two words were manipulated orthogonally, in order to obtain two lists of 40 word pairs each (see Table 1). High and low-frequency words were selected according to (cf. [38]). Half of the stimuli were of high-frequency (>92 occurrences per million, average 447; SD 326,3; Range 97–1469) and half were of low-frequency (<92 occurrences per million, average 43; SD 19,4; Range 25–87). 40 pairs of words were semantically related (SR) and half were semantically unrelated (SU) (see S1 Appendix). Semantic relatedness was determined via 7-point Likert scale questionnaire administered to 33 independent participants (mean age = 21.4 years, range = 20–23 years, SD = 0.8, 30 females) who did not take part in the main experiments. We established that pairs of words receiving a rate of 4.5 or higher were considered semantically related (e.g., *PANE-VINO; BREAD-WINE;* mean rate = 5.8, SD = 1.2; range = 2.5–7.0). Pairs of words receiving a rate of 3 or lower were considered semantically unrelated, e.g., *RIVA-ARTE; SHORE-ART*; mean rate = 1.7, SD = 1.2; range = 1.0–5.3) (see Table 2). The word pairs did not involve synonyms or antonyms.

**Table 1:** Example of stimulus manipulation used. HF and LF indicate high- and low-frequency words, respectively, whereas SR and SU refer to semantically related and semantically unrelated word pairs. The English translation of the stimuli is shown in brackets.

	SR (40 pairs)	SU (40 pairs)
**HF W1 – HF W2**	*SALE – PEPE* (SALT – PEPPER)	*IDEA – CARNE* (IDEA – MEAT)
**LF W1 – LF W2**	*PERA – MELA* (PEAR – APPLE)	*ASSO – CERA* (ACE – WAX)
**HF W1 – LF W2**	*LUCE – PALO* (LIGHT – POLE)	*LETTO – PAGA* (BED – PAYS)
**LF W1 – HF W2**	*FALCO – VOLO* (HAWK – FLIGHT)	*SITO – PACE* (SITE – PEACE)

**Table 2:** Main descriptive statistics across all independent variables used in Experiment 1 and 2. Mean, standard deviations and range values are reported for length, frequency and semantic relatedness for semantically related (SR) and semantically unrelated (SU) word pairs.

	SR pairs (N = 40)		SU pairs (N = 40)	
	W1	W2	W1	W2
Length	4.6 (0.5), 4-5	4.5 (0.5), 4-5	4.4 (0.5), 4-5	4.5 (0.5), 4-5
Frequency	229.4 (260), 25-876	231.7 (353.6), 25-1469	262.3 (302.1), 25-1058	255 (314.9), 26-1219
Semantic relatedness	5.8 (1.2), 2.5-7		1.7 (1.2), 1-5.3	

### Software and apparatus

The Experiment Builder software (SR Research Ltd., Mississauga, ON, Canada) was used for programming and running the experiments. In order to ensure the fixation stability and retinal position of the stimuli prior to their presentation on the screen, eye movements were recorded via an SR Research Ltd. EyeLink 1000 Plus eye-tracker, sampling at 1000 Hz. Participants were seated at around 57 cm from a 27-inch LED monitor (1366 × 768 pixels, 60 Hz), with their head movement restricted by a chin-rest. Vocal responses were recorded via a one-way microphone connected to an external sound card (M-track 22).

### Experimental procedure

At the beginning of each experiment, a standard nine-point calibration and validation procedure was conducted. Each trial began with a fixation cross (subtending a visual angle of 0.5°) displayed on the left side of the screen. When fixation was maintained for at least 250 ms, the cross disappeared and a pair of words (W1 and W2) appeared for 150 ms. The fixation cross was positioned between the second and the third letters of W1, corresponding to the optimal viewing position [39].

The order of presentation of the word pairs was pseudo-randomized across participants, with W1 displayed in the fovea and W2 in the parafovea. Words were presented in the Courier New, a monospaced font ensuring equal center-to-center letter spacing, and each letter subtended 0.5° of visual angle. The total horizontal extent of the word pairs ranged from 7.5° (both words 4 letters) to 9.5° (both words 5 letters).

In 50% of trials (20 SR and 20 SU), a red asterisk (the probe) appeared immediately (0 ms delay) after the onset of the participant’s vocal response. In Experiment 1, the probe appeared just to the right of W2 (7 pixels/ 0.18°), and in Experiment 2, the probe appeared 7 pixels/ 0.18° above W1. Participants were instructed to (1) read both words aloud and (2) press a button on the button-box as quickly as possible when the probe appeared, with both speed and accuracy being emphasized. Each trial ended with a response screen, followed by a 3000 ms intertrial interval. The following dependent measures were recorded: 1) Vocal response times (vRTs) to W1; 2) Accuracy in reading W1 and W2; 3) Probe detection accuracy and 4) probe reaction times (pRTs), defined as the interval from probe onset to button press. Notably, the procedure allowed pRTs to be measured independently of vRTs.

### Data cleaning and statistical procedure

As reported in the previous section, the following dependent measures underwent statistical analysis: vRTs to W1; accuracy in reading W2; probe detection accuracy and pRTs. We excluded from the analysis the participants who, because of low accuracy (below 30%) and recording failures, did not have data values in at least one of the experimental conditions (5.4%). We also excluded participants who detected fewer than half of the probe trials (i.e., missing ≥ 10 trials) in at least one experimental condition (8%). Furthermore, by visual inspection of each trial, we excluded trials containing saccades directed towards the parafoveal word. Analyses for W2 accuracy, probe accuracy, and pRTs included only trials where W1 was correctly named. vRTs were analyzed only for trials in which both W1 and W2 were correctly read. Outliers (vRTs and pRTs > 2.5 SDs <from each participant’s mean) were removed.

We applied mixed-effects models to the dependent variables reported above by using the 2.6.44 Jamovi version and the library package GAMLj3. Linear mixed-effects models were fitted to vocal response time data and probe response time data, with W1 frequency **(**high- vs. low-frequency), W2 frequency (high- vs. low-frequency), and semantic relatedness (unrelated vs. related) as fixed factors, and subjects as random intercept. The model assumed Gaussian residuals and used Satterthwaite’s approximation for degrees of freedom. Bonferroni post-hoc was adopted for exploring the interactions. Generalized mixed-effects models (logistic) were fitted to binary accuracy data – accuracy on W2 and probe accuracy – with W1 frequency, W2 frequency, and semantic relatedness entered as fixed factors, and subjects entered as random factors. The model used a logit link function and Wald confidence intervals.

## Results

### Experiment 1 – probe to the right of parafoveal word

Four participants were excluded from the analysis: one due to low accuracy in reading W2 (>30%) and three due to probe detection performance ≤10 trials in at least one experimental condition. Full descriptive statistics for all dependent variables across all experimental conditions are reported in S2 Appendix.

#### Accuracy in reading W2.

The generalized linear mixed-effects model converged successfully, and the model fit was adequate (Conditional *R²* = 0.219; Marginal *R²* = 0.097; *χ²*/df = 0.98), indicating that both fixed and random effects contributed meaningfully to explaining accuracy variance.

Overall mean accuracy was 96% for W1 (range = 84–100%) and 68% for W2 (range = 44–94%). The analysis revealed significant main effects of W1 frequency (*χ²*(1) = 6.81, *p* = 0.009), W2 frequency (*χ²*(1) = 10.12, *p* = 0.001), and semantic relatedness (*χ²*(1) = 96.91, *p* < 0.001). Accuracy was higher for high- vs. low-frequency words (W1: 73% vs 68%; *β* = –0.25, *SE* = 0.09, *OR* = 0.78, *p* = 0.009; W2: 74% vs 68%; *β* = –0.30, *SE* = 0.09, *OR* = 0.74, *p* = 0.001). Conversely, semantic relatedness increased the likelihood of a correct response (80% vs 60%; *β* = 0.93, *SE* = 0.09, *OR* = 2.54, *p* < 0.001).

Significant interactions were also observed for W1 × W2 frequency (*χ²*(1) = 12.00, *p* < .001), W2 frequency × semantic relatedness (*χ²*(1) = 28.74, *p* < .001), and the three-way interaction (*χ²*(1) = 27.02, *p* < .001). The W1 × semantic relatedness interaction was not significant (*p* = 0.35).

**Fig 1:**
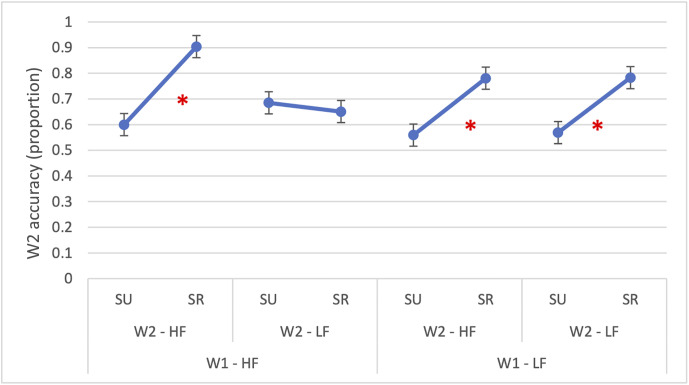
Experiment 1: Accuracy in reading W2. The figure illustrates the significant three-way interaction in accuracy between W1 frequency (HF = high-frequency; LF = low-frequency), W2 frequency (HF = high-frequency; LF = low-frequency) and semantic relatedness (SR = semantically related; SU = semantically unrelated). The asterisk (*) indicates statistically significant differences (p < 0.05). Error bars represent the standard error.

The three-way interaction (*β* = 1.96, *SE* = 0.38, *OR* = 7.11, *p* < 0.001) is reported in Fig 1 and it revealed that the joint facilitation of frequency and semantic relatedness was particularly pronounced when both words were high-frequency and semantically related. The random intercept for subjects accounted for little variability (Var = 0.52, SD = 0.72, ICC = 0.14), indicating that about 14% of variance in response accuracy was due to between-subject differences.

#### Vocal reaction times on W1.

The model converged successfully, the conditional and marginal R² were 0.53 and 0.02, respectively, and significant main effects were found for W1 frequency [F(1,1591) = 5.61, p = 0.018], W2 frequency [F(1,1592) = 11.34, p < 0.001], and semantic relatedness [F(1,1592) = 19.02, p < 0.001]. Parameter estimates revealed that RTs were slower for low-frequency words compared to high-frequency ones for both W1 (766 vs 745 ms; β = 21.67, SE = 9.15, p = 0.018) and W2 (771 vs 740 ms; β = 30.83, SE = 9.16, p < 0.001). In addition, semantically related pairs yielded shorter RTs than unrelated pairs (735 vs. 775; β = –40.06, SE = 9.19, p < 0.001).

**Fig 2:**
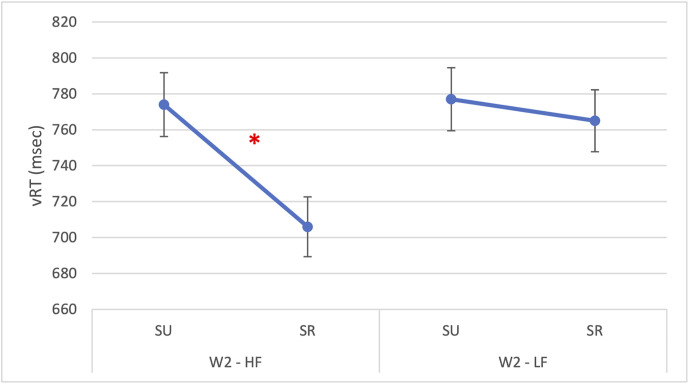
Experiment 1: Vocal reaction times on W1. The figure illustrates a significant interaction in vocal reaction times on W1 between W2 frequency (HF = high-frequency; LF = low-frequency) and semantic relatedness (SR = semantically related; SU = semantically unrelated). The asterisk (*) indicates statistically significant differences (p < 0.05). Error bars represent the standard error.

Importantly, the interaction between W2 frequency and semantic relatedness was also significant [F(1,1592) = 9.78, p = 0.002] indicating that the semantic facilitation effect was stronger for high-frequency W2 items (β = 57.31, SE = 18.32, p = 0.002), as represented in Fig 2. All other two-way and three-way interactions were non-significant (p*s* > 0.32).

The random intercept for subjects accounted for moderate variability (Var = 35,586, SD = 189; ICC = 0.52), indicating that about half of the variance in RTs was attributable to between-subject differences.

#### Accuracy in probe detection.

The model successfully converged, the model fit was satisfactory (Conditional *R²* = .211; Marginal *R²* = .124; *χ²*/df = 0.92), indicating that both fixed and random effects accounted for meaningful variance in accuracy. Significant main effects emerged for W1 frequency (*χ²*(1) = 4.06, *p* = 0.044), with higher accuracy for high- vs. low-frequency words (88% vs 83%; *β* = –0.35, *SE* = 0.17, *OR* = 0.71, *p* = 0.044) and semantic relatedness (*χ²*(1) = 19.05, *p* < .001) that increased the odds of a correct response (89% vs 80%; *β* = 0.75, *SE* = 0.17, *OR* = 2.11, *p* < 0.001). W2 frequency did not reach significance (*p* = 0.132).

**Fig 3:**
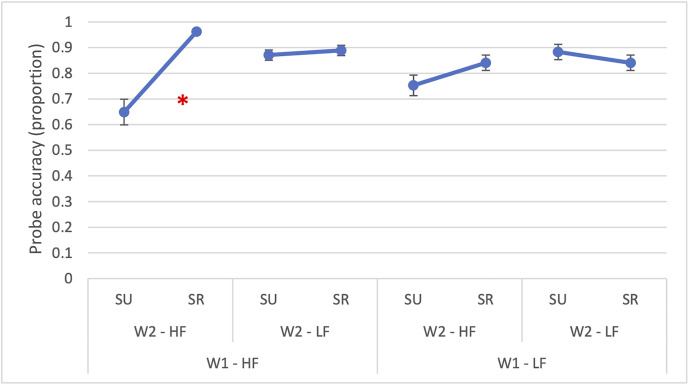
Experiment 1: Accuracy for probe detection placed to the right of the parafoveal word. The figure illustrates the significant three-way interaction in probe detection accuracy between W1 frequency (HF = high-frequency, LF = low-frequency), W2 frequency (HF = high-frequency, LF = low-frequency) and semantic relatedness (SR = semantically related; SU = semantically unrelated). The asterisk (*) indicates statistically significant differences (p < 0.05). Error bars represent the standard error.

The two-way interaction between W1 × semantic relatedness (*χ²*(1) = 14.46, *p* < 0.001) and between W2 × semantic relatedness (*χ²*(1) = 24.07, *p* < 0.001) were statistically significant. Also, the three-way interaction among W1 frequency, W2 frequency and semantic relatedness was significant (*χ²*(1) = 5.20, *p* = 0.023). The interaction (*β* = 1.56, *SE* = 0.68, *OR* = 4.76, *p* = 0.023) is reported in Fig 3 and it indicated that the joint effect of word frequency and semantic relatedness on probe accuracy was most pronounced when both words were high-frequency and semantically related.

The random intercept for subjects showed moderate variability (Var = 0.36, SD = 0.60, ICC = 0.10), suggesting that approximately 10% of the variance in probe accuracy was attributable to between-subject differences.

#### Reaction times for probe detection.

**Fig 4:**
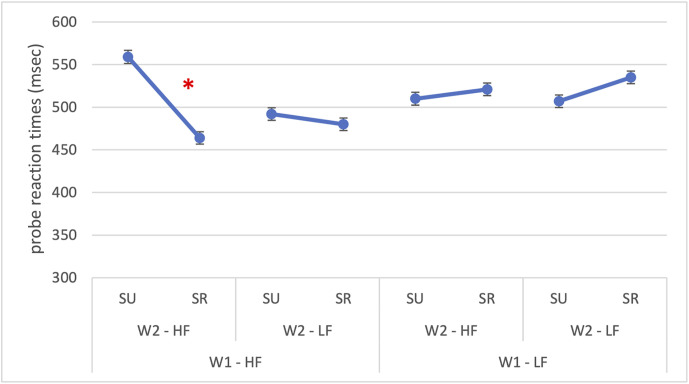
Experiment 1: Reaction times for probe detection placed to the right of the parafoveal word. The figure illustrates the significant three-way interaction in probe detection reaction times between W1 frequency (HF = high-frequency; LF = low-frequency), W2 frequency (HF = high-frequency; LF = low-frequency) and semantic relatedness (SR = semantically related; SU = semantically unrelated). The asterisk (*) indicates statistically significant differences (p < 0.05). Error bars represent the standard error.

The model successfully converged (*N* = 1049 observations, 32 participants; optimizer = *bobyqa*), the conditional and marginal R² were 0.371 and 0.052, respectively, indicating that both fixed and random effects together explained 37.1% of the variance in RTs. Significant main effects were found for W1 frequency, *F*(1,1010) = 10.06, *p* = 0.002, and semantic relatedness, *F*(1,1010) = 8.06, *p* = .005. The main effect of W2 frequency was not significant (*p* = 0.096). Probe reaction times were slower for low-frequency W1 compared to high-frequency W1 (518 vs 499 ms; *β* = 19.1, *SE* = 6.02, *p* = 0.002), and semantic relatedness facilitated responses (500 vs 517 ms; *β* = –17.1, *SE* = 6.02, *p* = 0.005). However, these effects were further modulated by several significant interactions: W1 × W2 frequency, *F*(1,1011) = 6.51, *p* = 0.011; W1 frequency × semantic relatedness, *F*(1,1011) = 36.58, *p* < 0.001; W2 frequency × Semantic relatedness, *F*(1,1011) = 17.40, *p* < 0.001; and the three-way interaction W1 frequency × W2 frequency × Semantic relatedness, *F*(1,1011) = 7.83, *p* = 0.005. The significant three-way interaction (*β* = –67.4, *SE* = 24.1, *p* = 0.005) is reported in Fig 4 and it revealed that facilitation patterns were strongest when both words were high-frequency and semantically related.

The random intercept for subjects (Var = 4739, SD = 68.8, ICC = 0.34), indicated that about one-third of the variability in RTs was attributable to between-subject differences.

#### Discussion of Experiment 1 results.

The results of Experiment 1 showed that participants were more accurate in reading the parafoveal word (W2) when the foveal and the parafovea words were of high-frequency and when a semantic relatedness existed between the two words. Similarly, vocal reaction times for W1 were faster when W2 was of high-frequency and when it was semantically related to W1. These findings are consistent with both the parafoveal preview effect (PPE) and the parafoveal-on-foveal (PoF) effect previously reported in the literature (see [33,34]). Importantly, we also found that probe detection was both faster and more accurate when both words were high-frequency and semantically related.

### Experiment 2 – probe above the foveal word

Six participants were excluded from the analyses due to low accuracy on W2 (three participants with W2 accuracy <30%) or an insufficient number of valid trials for probe detection (three participants with ≤10 trials per condition). Full descriptive statistics for all dependent variables across all experimental conditions are reported in S2 Appendix.

#### Accuracy in reading W2.

Overall accuracy on W1 was 96% (range = 86–100%) while accuracy on W2 was 63% (range = 38–93%). Model fit was adequate (conditional R² = 0.19; marginal R² = 0.09), the overdispersion was minimal (χ²/df = 0.98), and significant main effects were found for W1-F [χ²(1) = 7.45, *p* = 0.006], W2-F [χ²(1) = 17.79, *p* < 0.001], and semantic relatedness [χ²(1) = 59.95, *p* < 0.001]. Responses were less accurate for LF words **(**W1: 69% vs 63%; β = −0.25, *SE* = 0.09, *z* = −2.73, *p* = 0.006; W2: 70% vs 61%; β = −0.38, *SE* = 0.09, *z* = −4.22, *p* < 0.001). Accuracy was higher for semantically related pairs (73% vs 58%; β = 0.70, *SE* = 0.09, *z* = 7.74, *p* < 0.001).

There were significant two-way interactions between W1 frequency and W2 frequency [χ²(1) = 22.75, *p* < 0.001], and W2 frequency and semantic relatedness [χ²(1) = 64.64, *p* < 0.001]. The three-way interaction among W1 frequency, W2 frequency and semantic relatedness was also significant [χ²(1) = 4.71, *p* = 0.030].

**Fig 5:**
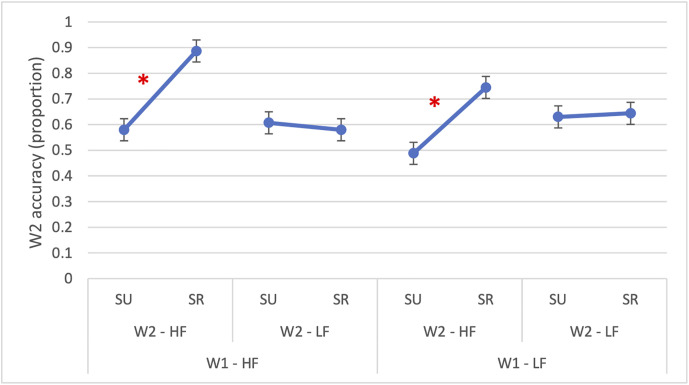
Experiment 2: Accuracy in reading W2. The figure illustrates the significant three-way interaction in accuracy between W1 frequency (HF = high-frequency; LF = low-frequency), W2 frequency (HF = high-frequency; LF = low-frequency) and semantic relatedness (SR = semantically related; SU = semantically unrelated). The asterisk (*) indicates statistically significant differences (p < 0.05). Error bars represent the standard error.

The three-way interaction (β = 0.78, *SE* = 0.36, *z* = 2.17, *p* = .030; OR = 2.19) is reported in Fig 5 and it revealed an increase in W2 accuracy when there is a semantic relatedness between the couple, especially when W2 is high-frequency.

Random effects showed low between-subject variance (σ² = 0.40; SD = 0.64; ICC = 0.11), indicating that about 11% of total variance in accuracy was attributable to subject-level differences.

#### Vocal reaction times on W1.

The model showed good overall fit (conditional R² = 0.54; marginal R² = 0.03), and significant main effects emerged for W1 frequency, [*F*(1, 1542) = 31.79, *p* < 0.001], W2-F [*F*(1, 1542) = 7.96, *p* = 0.005], and semantic relatedness [*F*(1, 1543) = 21.03, *p* < 0.001]. Responses were slower for LF compared to HF words for both positions [W1: 752 vs. 710 ms; β = 42.30, *SE* = 7.50, *t*(1542) = 5.64, *p* < 0.001; W2: 742 vs. 720 ms; β = 21.16, *SE* = 7.50, *t*(1542) = 2.82, *p* = 0.005). Responses were also fas*t*er for semantically rela*t*ed pairs compared to unrelated ones (748 vs. 714 ms; β = −34.53, *SE* = 7.53, *t*(1543) = −4.59, *p* < 0.001).

**Fig 6:**
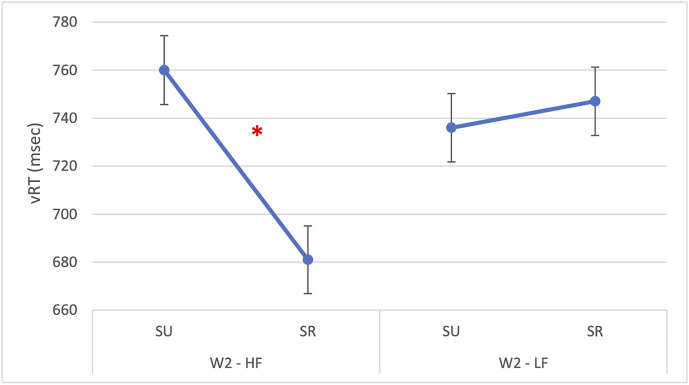
Experiment 2: Vocal reaction times on W1. The figure illustrates a significant interaction in vocal reaction times on W1 between W2 frequency (HF = high-frequency; LF = low-frequency) and semantic relatedness (SR = semantically related; SU = semantically unrelated). Asterisk (*) indicates statistically significant differences (*p* < 0.05). The bars indicate the standard error.

A significant interaction between W2 frequency and semantic relatedness was found [*F*(1, 1542) = 36.35, *p* < 0.001]. The interaction is reported in Fig 6 and it revealed that the semantic facilitation effect was larger when the second word had HF (β = 90.54, *SE* = 15.02, *t*(1542) = 6.03, *p* < 0.001). No o*t*her interactions were statistically significant (all *ps* > 0.05). Random effects indicated substantial variance across subjects (σ² = 24,109; SD = 155), with an intraclass correlation of 0.53, suggesting that about half of the total variance was attributable to between-session differences.

#### Accuracy in probe detection.

The model showed good convergence and explained a moderate portion of variance (marginal R² = 0.117; conditional R² = 0.176). There were significant main effects of W1 frequency (χ²(1) = 7.54, p = 0.006); W2 frequency (χ²(1) = 25.76, p < 0.001), and semantic relatedness (χ²(1) = 5.57, p = .018; OR = 1.47). Accuracy was higher for HF-W1 (HF:87% vs LF: 81%), LF-W2 (HF:78% vs LF: 89%) and semantically related couples (SR:87% vs SU: 82%). A significant interaction emerged between W1 frequency and semantic relatedness (χ²(1) = 14.11, p < 0.001). For HF-W1, semantic relatedness strongly increases accuracy (from mean = 0.8 to mean = 0.92). Conversely, for LF-W1, semantic relatedness actually reduces accuracy (from mean = 0.83 down to mean = 0.79). Also the interaction between W2 frequency and semantic relatedness was significant (χ²(1) = 13.44, p < 0.001), revealing that only for HF-W2 relatedness dramatically boosts accuracy (from mean = 0.68 to mean = 0.85).

**Fig 7:**
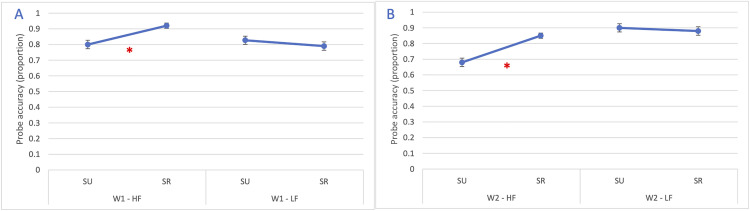
Experiment 2: Accuracy for probe detection placed above the foveal word. The figure illustrates two statistically significant interactions: (A) the interaction between W1 frequency (HF = high-frequency; LF = low-frequency) and semantic relatedness (SR = semantically related; SU = semantically unrelated); and (B) the interaction between W2 frequency (HF = high-frequency; LF = low-frequency) and semantic relatedness (SR = semantically related; SU = semantically unrelated). The asterisk (*) indicates statistically significant differences (p < 0.05). Error bars represent the standard error.

The three-way interaction among the two frequency variables and semantic relatedness did not reach significance (χ²(1) = 2.83, p = 0.09). The random intercept variance for subjects was 0.24 (SD = 0.49), suggesting small interindividual variability. The results of the two significant interactions are reported in Fig 7.

#### Reaction times for probe detection.

The model showed good convergence and explained a substantial proportion of variance (conditional R² = 0.49; marginal R² = 0.05). Significant main effects were observed for semantic relatedness [F(1, 998) = 21.48, p < 0.001] with shorter reaction times for semantically related pairs than for unrelated ones (472 vs 495 ms; β = −23.47, SE = 5.06, t(998) = −4.63, p < 0.001), whereas the main effect of W1or W2 frequency were not statistically significant [p*s* > 0.1].

There were significant two-way interactions between W1 frequency and W2 frequency [F(1, 998) = 5.43, p = 0.020], W1 Frequency and Semantic Relatedness [F(1, 999) = 53.23, p < 0.001], and W2 Frequency and Semantic Relatedness [F(1, 998) = 14.55, p < 0.001].

**Fig 8:**
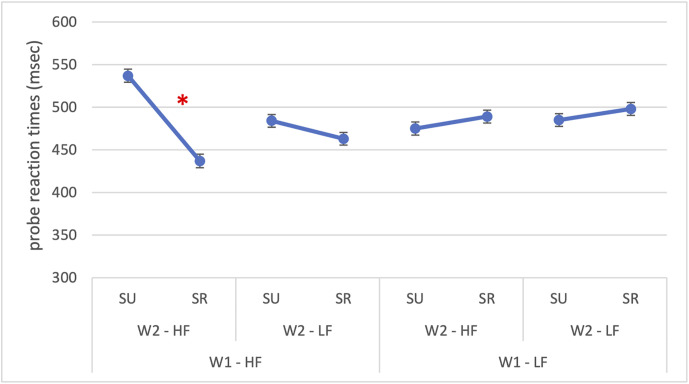
Experiment 2: Reaction times for probe detection placed above the foveal word. The figure illustrates the significant three-way interaction in probe detection reaction times between W1 frequency (HF = high-frequency; LF = low-frequency), W2 frequency (HF = high-frequency; LF = low-frequency) and semantic relatedness (SR = semantically related; SU = semantically unrelated). The asterisk (*) indicates statistically significant differences (p < 0.05). Error bars represent the standard error.

Importantly, the three-way interaction between W1 frequency, W2 frequency, and semantic relatedness was also significant, F(1, 998) = 16.45, p < 0.001. The three-way interaction (β = −82.25, SE = 20.28, t(998) = −4.06, p < 0.001) revealed that the effect of semantic relatedness depended jointly on the frequencies of both words: facilitation for related pairs was strongest when both words were of high-frequency, and weakest (or reversed) when frequencies were low, as shown in Fig 8.

Random effects suggested moderate variability across sessions (σ² = 5633; SD = 75.1), with an intraclass correlation coefficient (ICC) of.46, indicating that approximately half of the total variance in RTs was attributable to between-session differences.

#### Discussion of Experiment 2 results.

Consistent with Experiment 1, accuracy in reading the parafoveal word was higher when the two words were of high-frequency and when the two words were semantically related. Likewise, vocal reaction times revealed faster naming of the foveal word when W2 was of high-frequency and semantically related to the W1. Crucially, and in line with the results of Experiment 1, both accuracy and reaction times for probe detection were enhanced when the W1 and W2 were of high-frequency and semantically related. Conversely, when the foveal word was of low-frequency, no semantic facilitation effects were observed in either accuracy or reaction times, suggesting a reduced influence of parafoveal semantic information under conditions of increased foveal processing demands.

## Discussion

In this study, we investigated whether semantic processing in the parafovea can occur in parallel with foveal word processing, as well as how spatial attentional resources are distributed during brief, simultaneous word presentations.

Building on previous research using the Rapid Parallel Visual Presentation (RPVP) paradigm [33–35] we conducted two experiments in which participants read aloud two words—one centrally located and one in parafoveal vision—and subsequently performed a non-linguistic probe detection task. This design enabled us to jointly assess how lexical-semantic variables affect attentional load and spatial allocation of attention.

In terms of reading performance, we observed two significant main effects: W1 frequency and semantic relatedness. Indeed, participants were more accurate in reading the parafoveal word (W2) and faster in naming the foveal word (W1) when W1 was a high-frequency word and also when it was semantically related to W2. These two effects did not interact. These results are consistent with the predictions of parallel models of reading (e.g., [14–16]), which posit that multiple words can be processed simultaneously and that attention is distributed across a gradient extending beyond the point of fixation. Moreover, our findings align with parafoveal-on-foveal (PoF) effects [21,22,24], showing that the semantic properties of parafoveal words can influence the processing of foveal words.

Importantly, our study contributes novel evidence by coupling the RPVP paradigm with a non-linguistic probe detection task, which allowed us to directly test the attentional load and the spatial distribution of attention during reading-like tasks. In Experiment 1, the probe was positioned to the right of the parafoveal word; in Experiment 2 it appeared above the foveal word. Across both experiments, semantic facilitation in probe detection emerged reliably when W1 was high-frequency, and it was strongest when both words were high-frequency.

According to parallel models of reading (e.g., SWIFT [18]), if attention is spatially extended across the entire region occupied by the words, both in terms of spatial allocation and depth of semantic processing, then probe detection should: (i) benefit from semantic relatedness between the words, and (ii) show no difference based on the probe’s spatial position. Consistent with these predictions, we observed enhanced probe detection when W1 and W2 were of high-frequency and semantically related, regardless of probe location. This semantic facilitation in a non-linguistic task suggests that attention was allocated not only on the foveal word but also on the parafoveal word, supporting the idea of graded, overlapping attentional fields that can integrate visual and semantic information across multiple words [15,28]. These findings further reinforce claims that semantic processing is not confined to foveal vision and can occur rapidly even for words not yet fixated [16,20].

Crucially, the semantic advantage disappeared under conditions of high foveal load—when W1 was a low-frequency word—suggesting that attentional resources were more heavily taxed by the demands of foveal lexical access. This finding supports the foveal load hypothesis [26], and is consistent with serial attention models (e.g., [8,9,12,13]), which assume that parafoveal processing is constrained by the availability of attentional resources, which are primarily engaged with the fixated word. Under high load, the allocation of attention to the parafovea—and thus parafoveal semantic processing—appears to be limited. Nevertheless, the fact that semantic parafoveal processing was observed under low load conditions (see reading accuracy and vocal reaction times results) and that probe detection performance did not vary between spatial positions (Experiments 1 and 2) presents a challenge to strictly serial models. These results instead support a more flexible, dynamic allocation of attention, as proposed by the SWIFT model and other parallel frameworks [14,17], which allow for the simultaneous processing of multiple words to varying degrees depending on context and cognitive load.

Moreover, our findings contribute to ongoing controversies regarding the robustness of the foveal load effect (FLE). Although early work provided evidence for FLE [26], more recent studies have raised questions about its reliability and generalizability [30,31]. Our results confirm that foveal load can reduce semantic preview and attentional spread, yet they also demonstrate that such modulation is not all-or-none, and that parallel processing may still occur under favorable conditions, such as when the foveal word is of high-frequency.

A further possible speculation might help account for the results. Indeed, it is possible that the probe detection efficiency depends on the cognitive resources available immediately after the reading task: the presence of semantic facilitation makes more attentional resources available to detect the probe. According to this hypothesis, when the first task imposes a high cognitive load (i.e., low-frequency of W1 and no semantic relatedness) the probe should be less detectable compared to the condition of lower cognitive load (i.e., high-frequency and semantic relatedness between the two words). This effect due to overall cognitive load would be spatially independent, justifying the similar results obtained in terms of probe detection when presented on foveal or parafoveal locations.

One strength of our study lies in the use of a non-linguistic probe task as a proxy for attentional allocation. This method minimizes confounds associated with linguistic decision-making and provides a more direct behavioral measure of attentional deployment across the visual field. However, future studies should consider extending this paradigm by incorporating eye-tracking or EEG to further examine the temporal dynamics and neural correlates of parallel processing.

Finally, the present study has some limitations. Our mixed-effects models included random intercepts but not random slopes; although this modeling choice represents a limitation, the consistency of the observed effects across experiments and dependent measures supports their robustness. Furthermore, our design was limited to two-word displays with fixed spatial locations, real-world reading involves more complex syntactic and contextual constraints.

Future research should investigate whether similar effects can be observed in continuous text and whether parafoveal semantic processing generalizes to more naturalistic settings, potentially using gaze-contingent paradigms or more ecologically valid stimuli.

## Supporting information

S1 Appendix List of the stimuli used in Experiments 1 and 2. (DOCX)

S2 Appendix Main descriptive statistics for all dependent variables in Experiments 1 and 2. (DOCX)
