# Supplementary material for: Distribution of visuo-attentional resources while reading multiple words
Source: PLoS One. 2026 Feb 2;21(2):e0341917. doi: 10.1371/journal.pone.0341917 (PMC12863487; doi:10.1371/journal.pone.0341917)
Supplement: S1 Appendix — (DOCX) [file pone.0341917.s001.docx]

**S1 Appendix.**

**List of the stimuli used for the Experiments 1 and 2.**

Two lists of stimuli composed by 40 semantically unrelated (SU) word pairs and 40 semantically related (SR) word pairs. The frequency use was manipulated in order to obtain half of the stimuli of high-frequency use and half of low-frequency use.

| **List of stimuli** | **Category** | **Foveal word (W1)** | **W1 Length** | **W1 Frequency use** | **Parafoveal word (W2)** | **W2 Length** | **W2 Frequency use** |
| --- | --- | --- | --- | --- | --- | --- | --- |
| bolla canoa | SU | bolla | 5 | 27 | canoa | 5 | 26 |
| dado pizza | SU | dado | 4 | 41 | pizza | 5 | 27 |
| paio urlo | SU | paio | 4 | 367 | urlo | 4 | 81 |
| sala molla | SU | sala | 4 | 460 | molla | 5 | 31 |
| cura arena | SU | cura | 4 | 375 | arena | 5 | 35 |
| letto paga | SU | letto | 5 | 511 | paga | 4 | 35 |
| faro perla | SU | faro | 4 | 39 | perla | 5 | 30 |
| asino urna | SU | asino | 5 | 27 | urna | 4 | 56 |
| topo mago | SU | topo | 4 | 69 | mago | 4 | 86 |
| tipo palco | SU | tipo | 4 | 871 | palco | 5 | 87 |
| mamma ponte | SU | mamma | 5 | 491 | ponte | 5 | 166 |
| tubo cassa | SU | tubo | 4 | 74 | cassa | 5 | 226 |
| inno clima | SU | inno | 4 | 36 | clima | 5 | 236 |
| capra frase | SU | capra | 5 | 31 | frase | 5 | 272 |
| ritmo rete | SU | ritmo | 5 | 254 | rete | 4 | 436 |
| sito pace | SU | sito | 4 | 28 | pace | 4 | 401 |
| leva segno | SU | leva | 4 | 80 | segno | 5 | 530 |
| serie bene | SU | serie | 5 | 708 | bene | 4 | 548 |
| fondo prova | SU | fondo | 5 | 613 | prova | 5 | 647 |
| zona libro | SU | zona | 4 | 818 | libro | 5 | 1004 |
| tela aceto | SU | tela | 4 | 97 | aceto | 5 | 26 |
| luna esca | SU | luna | 4 | 181 | esca | 4 | 27 |
| cera asso | SU | cera | 4 | 25 | asso | 4 | 28 |
| pugno sugo | SU | pugno | 5 | 168 | sugo | 4 | 29 |
| agio lente | SU | agio | 4 | 57 | lente | 5 | 31 |
| arco urto | SU | arco | 4 | 86 | urto | 4 | 31 |
| esame culla | SU | esame | 5 | 334 | culla | 5 | 33 |
| penna scia | SU | penna | 5 | 80 | scia | 4 | 35 |
| atto vena | SU | atto | 4 | 548 | vena | 4 | 62 |
| tasto orso | SU | tasto | 5 | 25 | orso | 4 | 69 |
| pari dono | SU | pari | 4 | 34 | dono | 4 | 103 |
| molo tetto | SU | molo | 4 | 32 | tetto | 5 | 154 |
| latte nave | SU | latte | 5 | 152 | nave | 4 | 182 |
| dato radio | SU | dato | 4 | 470 | radio | 5 | 182 |
| idea carne | SU | idea | 4 | 1058 | carne | 5 | 232 |
| bando tono | SU | bando | 5 | 29 | tono | 4 | 352 |
| riva arte | SU | riva | 4 | 117 | arte | 4 | 622 |
| sete aria | SU | sete | 4 | 32 | aria | 4 | 774 |
| fine gente | SU | fine | 4 | 1024 | gente | 5 | 1052 |
| fauna sera | SU | fauna | 5 | 25 | sera | 4 | 1219 |
| tuffo nuoto | SR | tuffo | 5 | 27 | nuoto | 5 | 25 |
| mare alga | SR | mare | 4 | 630 | alga | 4 | 25 |
| voto lode | SR | voto | 4 | 606 | lode | 4 | 27 |
| zampa tigre | SR | zampa | 5 | 56 | tigre | 5 | 34 |
| ombra palma | SR | ombra | 5 | 265 | palma | 5 | 34 |
| corpo busto | SR | corpo | 5 | 876 | busto | 5 | 35 |
| toro corna | SR | toro | 4 | 33 | corna | 5 | 39 |
| porta varco | SR | porta | 5 | 753 | varco | 5 | 35 |
| orlo sarto | SR | orlo | 4 | 42 | sarto | 4 | 43 |
| pelo lupo | SR | pelo | 4 | 86 | lupo | 4 | 63 |
| sale pepe | SR | sale | 4 | 206 | pepe | 4 | 102 |
| nube fumo | SR | nube | 4 | 41 | fumo | 4 | 117 |
| buio notte | SR | buio | 4 | 126 | notte | 5 | 1152 |
| fiume lago | SR | fiume | 5 | 275 | lago | 4 | 131 |
| erba campo | SR | erba | 4 | 160 | campo | 5 | 983 |
| aroma caffè | SR | aroma | 5 | 38 | caffè | 5 | 164 |
| rissa lite | SR | rissa | 5 | 49 | lite | 4 | 99 |
| pane vino | SR | pane | 4 | 187 | vino | 4 | 283 |
| siepe fiore | SR | siepe | 5 | 27 | fiore | 5 | 410 |
| drago fuoco | SR | drago | 5 | 25 | fuoco | 5 | 609 |
| pera mela | SR | pera | 4 | 43 | mela | 4 | 66 |
| cibo mensa | SR | cibo | 4 | 236 | mensa | 5 | 25 |
| piede orma | SR | piede | 5 | 704 | orma | 4 | 25 |
| buca solco | SR | buca | 4 | 27 | solco | 5 | 27 |
| svago relax | SR | svago | 5 | 28 | relax | 5 | 27 |
| calza lino | SR | calza | 5 | 32 | lino | 4 | 29 |
| linea retta | SR | linea | 5 | 647 | retta | 5 | 47 |
| zucca dolce | SR | zucca | 5 | 33 | dolce | 5 | 53 |
| luce palo | SR | luce | 4 | 730 | palo | 4 | 56 |
| prete suora | SR | prete | 5 | 101 | suora | 5 | 86 |
| bocca naso | SR | bocca | 5 | 322 | naso | 4 | 131 |
| banca stato | SR | banca | 5 | 387 | stato | 5 | 1469 |
| foro buco | SR | foro | 4 | 32 | buco | 4 | 139 |
| pacco posta | SR | pacco | 5 | 56 | posta | 5 | 134 |
| cane gatto | SR | cane | 4 | 328 | gatto | 5 | 169 |
| salsa rosa | SR | salsa | 5 | 86 | rosa | 4 | 197 |
| falco volo | SR | falco | 5 | 33 | volo | 4 | 241 |
| crema viso | SR | crema | 5 | 107 | viso | 4 | 284 |
| torta festa | SR | torta | 5 | 67 | festa | 5 | 501 |
| cuore amore | SR | cuore | 5 | 672 | amore | 5 | 1153 |
