# Supplementary material for: Distribution of visuo-attentional resources while reading multiple words
Source: PLoS One. 2026 Feb 2;21(2):e0341917. doi: 10.1371/journal.pone.0341917 (PMC12863487; doi:10.1371/journal.pone.0341917)
Supplement: S2 Appendix — (DOCX) [file pone.0341917.s002.docx]

## S2 Appendix.

## Main descriptive statistics for all dependent variables in Experiments 1 and 2.

## The tables report estimated marginal means (EMM), standard errors (SE), and 95% confidence intervals (CI) for all statistically significant interactions. Values are shown as a function of word frequency (HF = high frequency; LF = low frequency) and semantic relatedness (SR = semantically related; SU = semantically unrelated) of the word pairs.

**Experiment 1 – probe to the right of parafoveal word**

## Accuracy in reading W2

| W1 Frequency | W2 Frequency | SemRel | EMM | SE | CI (lower-upper) |
| --- | --- | --- | --- | --- | --- |
| HF | HF | SU | 0.60 | 0.04 | [0.52 - 0.68] |
|  |  | SR | 0.90 | 0.02 | [0.86 - 0.94] |
|  | LF | SU | 0.69 | 0.04 | [0.61 - 0.76] |
|  |  | SR | 0.65 | 0.04 | [0.57 - 0.73] |
| LF | HF | SU | 0.56 | 0.04 | [0.47 - 0.64] |
|  |  | SR | 0.78 | 0.03 | [0.71 - 0.84] |
|  | LF | SU | 0.57 | 0.04 | [0.48 - 0.65] |
|  |  | SR | 0.78 | 0.03 | [0.71 - 0.84] |

## Vocal reaction times on W1 (ms)

| W2 Frequency | SemRel | EMM | SE | CI (lower-upper) |
| --- | --- | --- | --- | --- |
| HF | SU | 774 | 34.8 | [704 - 845] |
|  | SR | 706 | 34.3 | [636 - 775] |
| LF | SU | 777 | 34.7 | [706 - 847] |
|  | SR | 765 | 34.6 | [695 - 835] |

## Accuracy in probe detection

| W1 Frequency | W2 Frequency | SemRel | EMM | SE | CI (lower-upper) |
| --- | --- | --- | --- | --- | --- |
| HF | HF | SU | 0.65 | 0.05 | [0.55 - 0.73] |
|  |  | SR | 0.96 | 0.01 | [0.92 - 0.98] |
|  | LF | SU | 0.87 | 0.03 | [0.80 - 0.92] |
|  |  | SR | 0.89 | 0.03 | [0.83 - 0.93] |
| LF | HF | SU | 0.75 | 0.04 | [0.67 - 0.82] |
|  |  | SR | 0.84 | 0.03 | [0.77 - 0.89] |
|  | LF | SU | 0.88 | 0.03 | [0.82 - 0.93] |
|  |  | SR | 0.84 | 0.03 | [0.77 - 0.89] |

## Reaction times for probe detection (ms)

| W1 Frequency | W2 Frequency | SemRel | EMM | SE | CI (lower-upper) |
| --- | --- | --- | --- | --- | --- |
| HF | HF | SU | 559 | 15.5 | [528 - 590] |
|  |  | SR | 464 | 14.5 | [435 - 493] |
|  | LF | SU | 492 | 14.7 | [463 - 522] |
|  |  | SR | 480 | 14.7 | [451 - 510] |
| LF | HF | SU | 510 | 15.1 | [480 - 540] |
|  |  | SR | 521 | 14.8 | [491 - 551] |
|  | LF | SU | 507 | 14.7 | [478 - 536] |
|  |  | SR | 535 | 14.8 | [505 - 564] |

# Experiment 2 – probe above the foveal word

## Accuracy in reading W2

| W1 Frequency | W2 Frequency | SemRel | EMM | SE | CI (lower-upper) |
| --- | --- | --- | --- | --- | --- |
| HF | HF | SU | 0.58 | 0.04 | [0.50 - 0.66] |
|  |  | SR | 0.89 | 0.02 | [0.84 - 0.92] |
|  | LF | SU | 0.61 | 0.04 | [0.53 - 0.68] |
|  |  | SR | 0.58 | 0.04 | [0.50 - 0.66] |
| LF | HF | SU | 0.49 | 0.04 | [0.41 - 0.57] |
|  |  | SR | 0.75 | 0.03 | [0.68 - 0.80] |
|  | LF | SU | 0.63 | 0.04 | [0.55 - 0.70] |
|  |  | SR | 0.64 | 0.04 | [0.57 - 0.71] |

## Vocal reaction times on W1 (ms)

| W2 Frequency | SemRel | EMM | SE | CI (lower-upper) |
| --- | --- | --- | --- | --- |
| HF | SU | 760 | 28.7 | [702 - 818] |
|  | SR | 681 | 28.2 | [623 - 738] |
| LF | SU | 736 | 28.5 | [678 - 794] |
|  | SR | 747 | 28.5 | [689 - 805] |

## Accuracy in probe detection

| W1 Frequency | SemRel | EMM | SE | CI (lower-upper) |
| --- | --- | --- | --- | --- |
| HF | SU | 0.80 | 0.03 | [0.74 – 0.85] |
|  | SR | 0.92 | 0.02 | [0.88 – 0.94] |
| LF | SU | 0.83 | 0.03 | [0.77 – 0.87] |
|  | SR | 0.79 | 0.03 | [0.74 – 0.84] |

| W2 Frequency | SemRel | EMM | SE | CI (lower-upper) |
| --- | --- | --- | --- | --- |
| HF | SU | 0.68 | 0.03 | [0.62 – 0.74] |
|  | SR | 0.85 | 0.02 | [0.80 – 0.89] |
| LF | SU | 0.90 | 0.02 | [0.86 – 0.93] |
|  | SR | 0.88 | 0.02 | [0.83 – 0.92] |

## Reaction times for probe detection (ms)

| W1 Frequency | W2 Frequency | SemRel | EMM | SE | CI (lower-upper) |
| --- | --- | --- | --- | --- | --- |
| HF | HF | SU | 537 | 15.4 | [506 - 568] |
|  |  | SR | 437 | 14.9 | [407 - 467] |
|  | LF | SU | 484 | 14.9 | [453 - 514] |
|  |  | SR | 463 | 14.8 | [433 - 493] |
| LF | HF | SU | 475 | 15.4 | [444 - 505] |
|  |  | SR | 489 | 15.1 | [459 - 520] |
|  | LF | SU | 485 | 14.9 | [455 - 515] |
|  |  | SR | 498 | 15.1 | [467 - 528] |
